# Microbial dysbiosis and predictive neuroactive functional profiles in paediatric neurogenic bladder: an exploratory machine learning-based approach

**DOI:** 10.3389/fcimb.2026.1875579

**Published:** 2026-08-26

**Authors:** Deepthi Ramya Ravindran, Sachit Anand, Ajay Verma, Jitendra Kumar Meena, Himalaya Kumar, Pankaj Hari, Sumit Aggarwal, Vineet Ahuja

**Affiliations:** 1 Department of Pediatric Surgery, All India Institute of Medical Sciences (AIIMS), New Delhi, India; 2 Division of Pediatric Nephrology, Department of Pediatrics, All India Institute of Medical Sciences (AIIMS), New Delhi, India; 3 Division of Descriptive Research, Indian Council of Medical Research, New Delhi, India; 4 Department of Gastroenterology, All India Institute of Medical Sciences (AIIMS), New Delhi, India

**Keywords:** 16S rRNA sequencing, dysbiosis, neurogenic bladder (NB), supervised machine learning models (SML), urinary microbiome and urobiome

## Abstract

**Background:**

Neurogenic bladder (NB) in children is associated with impaired bladder emptying, recurrent urinary tract infections (rUTIs), and progressive upper urinary tract deterioration. Although urinary microbiome dysbiosis has been increasingly recognised in NB, its neuroactive functional potential and relationship to disease severity remain poorly understood. We investigated urinary microbial composition and predicted neuroactive metabolic pathways and evaluated their ability to stratify disease severity using integrative multivariate and machine learning (ML) analyses.

**Methods:**

This cross-sectional study included 83 children comprising 39 patients with NB and 44 age- and sex-matched healthy controls. Patients with NB were stratified into Subgroup A (renal deterioration; *n* = 15) and Subgroup B (without renal deterioration; *n* = 23) based on glomerular filtration rate and renal scarring (DMSA). The urinary microbiome was characterised by 16S rRNA gene sequencing, and functional potential was inferred using PICRUSt2. A total of 25 literature-curated neuroactive MetaCyc pathways were analysed to generate a composite Neuroactive Dysbiosis Index (NDI). Associations between microbial taxa and predicted pathways were explored using correlation, hierarchical clustering, and network analyses. Partial least squares discriminant analysis (PLS-DA) and Random Forest (RF) models were used to identify discriminatory microbial signatures and evaluate classification performance.

**Results:**

NB exhibited marked urinary microbial dysbiosis accompanied by predicted shifts in neuroactive metabolic pathways. Disease-associated microbiomes demonstrated predictive enrichment of pathways involved in amino acid catabolism, polyamine metabolism, coenzyme A and phospholipid biosynthesis, one-carbon metabolism, and central energy metabolism, together with depletion of pathways associated with microbial metabolic homeostasis and neurotransmitter precursor biosynthesis. The NDI demonstrated a progressive increase with disease severity (Control < Subgroup B < Subgroup A), with significant differences between Subgroup A and controls (*p* = 0.004) and between Subgroup A and Subgroup B (*p* = 0.002). Network analysis revealed extensive reorganisation of genus–pathway interactions in deteriorated NB, whilst PLS-DA demonstrated clear functional separation between study groups. RF models achieved strong discrimination using predicted neuroactive pathways alone [area under the curve (AUC) = 0.882], which further improved when combined with microbial taxonomic features (AUC = 0.981), identifying phospholipid biosynthesis, coenzyme A metabolism, aromatic amino acid metabolism, and tRNA charging as the most informative discriminatory pathways.

**Conclusions:**

Paediatric NB is characterised by coordinated urinary microbial dysbiosis and alterations in predicted neuroactive functional pathways that parallel disease severity. Integrating microbial taxonomic composition with predicted functional profiles substantially improved disease classification, highlighting the potential of computationally inferred neuroactive microbial signatures for disease stratification. These findings provide new insights into the microbiome–neuroactive axis in paediatric NB.

## Graphical Abstract

**Figure:**
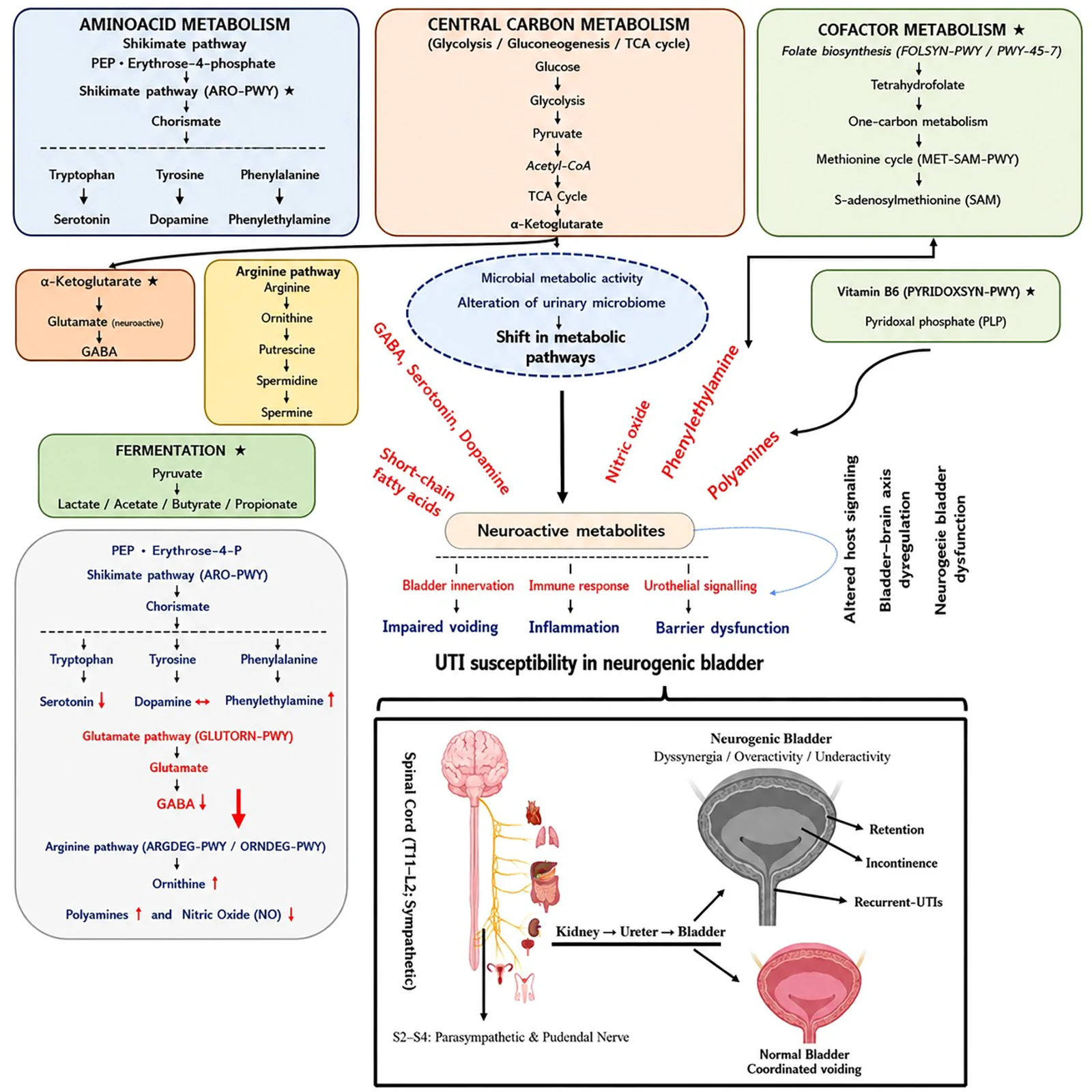

## Introduction

Paediatric neurogenic bladder (NB) is a major clinical concern, most commonly arising from congenital neurological conditions such as spina bifida (myelomeningocele), which accounts for the majority of childhood cases. Epidemiological evidence indicates that approximately 90%–95% of children with spina bifida develop some degree of bladder dysfunction (Sič et al., 2025). Other contributing conditions include cerebral palsy, spinal cord malformations, and acquired neurological injuries. Early-onset bladder dysfunction, if inadequately managed, can lead to progressive renal damage, recurrent urinary tract infections (rUTIs), and long-term morbidity (Meng et al., 2021). Surveillance data from the Center for Disease Control and Prevention (CDC) emphasise the importance of early diagnosis and structured bladder management strategies, particularly in children with spina bifida. Additionally, reports from the National Healthcare Safety Network (NHSN) highlight the increased susceptibility of patients with NB, especially those requiring catheterisation, to catheter-associated urinary tract infections (CAUTIs), further contributing to disease progression (Sager et al., 2022).

Traditionally, the urinary tract was considered sterile. However, advances in high-throughput sequencing technologies have led to the recognition of a distinct and dynamic urinary microbiome (Perez-Carrasco et al., 2021). Emerging evidence suggests that this microbial community plays a critical role in maintaining urothelial integrity, modulating immune responses, and influencing bladder function (Zhang et al., 2025). In disease states such as NB, alterations in microbial composition, termed dysbiosis, have been associated with rUTIs, inflammation, and impaired bladder function. However, most studies have focused primarily on taxonomic changes, with limited exploration of the functional metabolic potential of the urinary microbiome (Forster et al., 2020; Xie et al., 2020).

The urinary microbiome has emerged as an important modulator of bladder function, particularly in NB, where disruption of neural control is combined with microbial dysbiosis within the urinary tract (Forster and Pohl, 2019; Lane et al., 2022). These microbes are capable of producing or modulating neuroactive compounds, including γ-aminobutyric acid (GABA), serotonin, dopamine, and metabolites derived from tryptophan (Strandwitz, 2018). In NB, alterations in the composition and diversity of the urinary microbiome may disrupt these neuroactive pathways, contributing to abnormal bladder sensations and impaired detrusor activity (Lloyd-Price et al., 2019). For instance, reduced abundance of beneficial microbial taxa may lead to decreased GABA production, weakening inhibitory control over bladder reflexes and promoting detrusor overactivity (Douglas et al., 2020).

Additionally, urinary microbiota can influence host neurotransmitter systems through metabolites that interact with urothelial cells and local nerve endings. Tryptophan metabolism within the urinary tract can generate indole derivatives that modulate serotonin pathways, thereby affecting bladder signalling and coordination. Similarly, microbial interactions may influence dopaminergic signalling involved in central–peripheral communication (Strandwitz et al., 2019). Dysbiosis in NB is also associated with increased inflammation and altered epithelial integrity, which can further sensitise afferent nerves and exacerbate urinary symptoms such as urgency, frequency, and incontinence (Groah et al., 2023). Overall, the urinary microbiome acts as a local regulator of neuroactive pathways, and its imbalance in NB highlights a critical link between microbial networks and neurotransmitter-mediated bladder dysfunction.

Defects in the spinal cord disrupt neural pathways that regulate bladder function, leading to NB, characterised by detrusor overactivity and uninhibited contractions. This condition is driven by excessive release of excitatory neurotransmitters like acetylcholine (ACh), resulting in bladder hyperreflexia. These changes create environmental stress for the urinary microbiome, as indicated by the “Anna Karenina Principle” (AKP), where stressed microbial communities diverge from their normal state, affecting diversity and functionality. Adaptations may include enhanced biofilm formation, altered metabolism, and increased antimicrobial resistance, similar to observations in gut microbiome mechanisms, where physiological stress and inflammation lead to dysbiosis and functional changes in microbial communities (Ma, 2020; Yano et al., 2015).

Building on the observed interplay between urinary microbiome dysbiosis and neurotransmitter imbalance, this study was designed to characterise the neuroactive functional mechanisms of the urinary microbiome in NB. Specifically, we aimed to move beyond conventional taxonomic descriptions and investigate how microbial-derived neurotransmitter pathways (including GABA, tryptophan, serotonin, and dopamine metabolism) are altered across disease states, and the role these alterations contribute to bladder dysfunction. To achieve this, we implemented integrated analytics that combined 16S rRNA sequencing, predictive functional profiling by Picrust2, and advanced statistical and machine learning (ML) approaches to quantify neuroactive dysbiosis and identify key predictive microbial–pathway interactions. Furthermore, ML models were employed to evaluate the discriminative and predictive capacity of microbial and functional features, both independently and in combination. Collectively, this study highlights the urinary microbiome as a critical regulator of neurotransmitter-mediated bladder function and provides a foundation that integrates microbial and neurochemical modulation in paediatric NB populations.

## Methods

### Study design

This cross-sectional study was conducted in the Department of Pediatric Surgery at the All India Institute of Medical Sciences, New Delhi, India. Ethical approval was obtained from the Institutional Ethics Committee before initiation (Ref no. AIIMSA6090). All procedures adhered to institutional protocols and internationally accepted ethical standards for research involving human participants. Written informed consent was obtained from parents or legal guardians, and age-appropriate assent was taken from participating children.

### Study population

The study included 83 children. Among them, 39 children aged < 14 years were diagnosed with NB and were on regular follow-up at the outpatient department. Diagnosis of NB was established based on clinical evaluation, imaging findings, and urodynamic studies in accordance with standard criteria. Children in the NB group were excluded if they had an active urinary tract infection (defined as >10^5^ CFU/mL with symptoms), had received antibiotics within the preceding 3 months, or had any primary urological or systemic condition. In parallel, 44 healthy children matched for age and sex were enrolled as controls during the same time period. These participants were excluded if they had a history of renal disease, prior urinary tract infection, or abnormal findings on routine urine examination.

### Upper urinary tract evaluation and subgroup classification

As part of routine follow-up, upper urinary tract function was assessed using radionuclide imaging. Technetium-99m DTPA scans were used to estimate glomerular filtration rate (GFR), whilst technetium-99m DMSA scans were performed to detect renal cortical scarring, identified as areas of reduced tracer uptake or photpenic areas. These assessments were conducted at enrolment and subsequently on an annual basis. Based on the most recent imaging results, participants were categorised into subgroups. Children showing significant upper tract deterioration, defined as GFR below 60 mL/min/1.73 m² or evidence of renal scarring on DMSA, were assigned to Subgroup A. Those without such findings were classified as Subgroup B. The healthy controls formed Subgroup C.

### Urine sample collection and study workflow

Single-spot morning urine samples were collected from all participants to minimise circadian variability. For children able to void, midstream clean-catch urine samples (50 mL) were collected in sterile containers. For children with NB who could not void, urine was obtained through clean intermittent catheterisation under strict aseptic precautions. Notably, none of the participants had an indwelling catheter at the time of sample collection. Immediately after collection, samples were transported on ice, centrifuged to remove cellular debris, and stored at −80 °C until further analysis. Identical collection and processing protocols were followed across all patients to ensure consistency and reduce potential bias.

### Sample processing and DNA extraction

Following centrifugation, the pellet obtained from each sample was resuspended for DNA extraction. Genomic DNA was extracted using the Qiagen DNA isolation kit (Catalog #47104 Qiagen) according to the manufacturer’s instructions. DNA quality was assessed using a NanoDrop spectrophotometer, with an A_260_/A_280_ ratio between 1.8 and 2.0 considered acceptable. Extracted DNA was stored at −80 °C to preserve integrity until sequencing.

### DNA quality assessment and 16S rRNA sequencing

Sequencing was carried out at an external facility. DNA concentration and quality were further evaluated using a Qubit fluorometer before sequencing. The V_3_–V_4_ hypervariable regions of the bacterial 16S rRNA gene were targeted and sequenced using the Illumina NovaSeq 6000 platform, enabling high-throughput analysis. Amplification was performed using the forward primer 5′-CCTACGGGNGGCWGCAG-3′ and the reverse primer 5′-GACTACHVGGGTATCTAATCC-3′.

### Sequencing quality control and bioinformatics analysis

Raw paired-end sequencing data in FASTQ format were subjected to initial quality assessment using FastQC (v0.11.9). Summary reports of quality metrics, including Phred scores, GC content, read length distribution, and adapter contamination, were generated using MultiQC (v1.14). Based on these results, reads were filtered and trimmed to remove low-quality regions and residual adapters, retaining only high-quality sequences for downstream analysis. Subsequent analysis was performed using the DADA2 pipeline (v1.28) in R (v4.3.1). Error rates were modelled directly from the data, and identical reads were dereplicated within each sample. True biological sequences were inferred using probabilistic methods, and paired-end reads were merged based on overlap criteria, with inconsistent reads discarded. Chimeric sequences were identified and removed using a consensus-based approach. The final output consisted of an amplicon sequence variant (ASV) table representing exact sequences at single-nucleotide resolution. Taxonomic classification was performed using a naïve Bayesian classifier against the SILVA reference database, assigning taxonomy from phylum to genus level with defined confidence thresholds. All sequence data and taxonomic assignments were retained to ensure transparency and reproducibility.

### Methodology pipeline

In this study, we combined microbiome profiling with ML approaches to explore neuroactive functional alterations in the urinary microbiome associated with NB. Urine samples were analysed using 16S rRNA gene sequencing, specifically targeting the V3–V4 region. The raw sequencing data underwent standard preprocessing steps, including trimming of adapter sequences, removal of low-quality reads, and elimination of chimeric sequences.

High-quality reads were processed using a reference-based workflow, and taxonomic profiles were generated at the genus level using DADA2. To extend beyond taxonomy, functional potential was inferred using PICRUSt2, which enabled prediction of pathway-level abundances based on MetaCyc annotations. Particular emphasis was placed on neuroactive metabolic pathways. To ensure comparability across samples, all features were appropriately normalised to address compositional differences. Based on clinical status, samples were grouped into three categories: Subgroup A, Subgroup B, and Subgroup C (healthy controls), representing varying microbiome states. All functional pathways were annotated and classified according to the MetaCyc pathway database nomenclature.

### Neuroactive dysbiosis index

From the output of PICRUSt2, relevant neuroactive pathways were *a priori* selected based on established biological evidence from published literature describing microbial neurotransmitter metabolism, short-chain fatty acid biosynthesis, amino acid catabolism, oxidative stress responses, and neuroimmune signalling. Pathways involved in microbial homeostasis and production of neuroprotective metabolites (e.g., GABA-, tryptophan-, and SCFA-related pathways) were classified as beneficial, whereas pathways associated with amino acid degradation, oxidative stress, redox imbalance, and dysbiosis-associated metabolic adaptation were classified as stress-associated (Davila et al., 2013; Agus et al., 2018; Hamamah et al., 2022; Jiang et al., 2025; Koh et al., 2016a; Lyon et al., 2020; Magnúsdóttir et al., 2015; Strandwitz, 2018; Tofalo et al., 2019; Uceda et al., 2023). This classification was used to generate an exploratory composite Neuroactive Dysbiosis Index (NDI) for systems-level comparison across study groups.

For each sample, an NDI was calculated using the formula: log^2^ [(sum of stress-associated pathways + 1)/(sum of beneficial pathways + 1)], which accounts for zero values and stabilises variability.

Where necessary, NDI values were log_1_^0^-transformed to improve data distribution. Differences between control and disease groups were evaluated using the two-sided Wilcoxon rank-sum test, whilst effect sizes were estimated using Cliff’s delta (δ). Statistical significance was set at *p* < 0.05. For comparisons across multiple subgroups (A, B, and C), the Kruskal–Wallis test was applied, followed by pairwise Wilcoxon tests with Benjamini–Hochberg correction (FDR threshold *Q* = 0.05). To investigate pathway-level variation, the 25 most variable neuroactive pathways (based on variance across samples) were selected. These were standardised using *Z*-score transformation and subjected to hierarchical clustering (Euclidean distance and complete linkage). Additionally, neurotransmitter-specific pathway profiles were aggregated at the subgroup level, standardised, and clustered to identify patterns linked to disease severity.

### Correlation, clustering, and network analysis

Associations between microbial genera and predictive functional pathways were assessed using Spearman’s rank correlation to accommodate non-normal data distributions. The resulting correlation matrix was filtered to retain meaningful associations (|ρ| > 0.3 and *p* < 0.05), followed by correction for multiple testing using the Benjamini–Hochberg method. Correlation values were then *Z*-score normalised to enable comparison across features.

Hierarchical clustering (Euclidean distance and complete linkage) was applied to combined taxa–pathway datasets to identify co-association patterns. To further characterise these relationships, network analysis was performed in which microbial taxa and pathways were represented as nodes, and significant correlations were denoted as edges. Edge weights reflected correlation strength, whilst direction indicated positive or negative associations. Separate networks were constructed for each subgroup (A, B, and C) to highlight subgroup-specific interaction patterns. Chord diagrams were additionally used to summarise global connectivity between microbial taxa and neurotransmitter-related pathways. To ensure consistency, both pathway and taxonomic abundance data were log-transformed (log^10^[*X* + 1]) and standardised using *Z*-scores prior to analysis. The NDI was also recalculated using aggregated standardised pathway scores across the selected 25 neuroactive pathways, providing a composite measure of functional dysbiosis.

### ML-based feature prioritisation and biological interpretation

To investigate the discriminatory performance and biological relevance of urinary microbiome taxonomic profiles and PICRUSt2-predicted neuroactive functional pathways, an explainable ML approach was implemented.

Three independent feature matrices were constructed, comprising (i) microbial genus-level relative abundance, (ii) a curated panel of 25 PICRUSt2-predicted neuroactive pathways, and (iii) an integrated dataset combining both taxonomic and functional features.

Because of the limited cohort size, the complete dataset was analysed without partitioning into independent training and testing subsets. Microbial abundance and predicted pathway abundance data were transformed using log^10^(*X* + 1) followed by *Z*-score standardisation before downstream analyses. Separate ML analyses were performed using genus-level features, predicted neuroactive pathway features, and the integrated dataset to evaluate the relative discriminatory contribution of taxonomic, functional, and combined microbiome signatures.

Classification performance was initially evaluated using receiver operating characteristic (ROC) analysis, with area under the curve (AUC) calculated for genus-level, predicted pathway-level, and integrated combined feature datasets. One-vs-rest (OVR)-ROC analyses were subsequently performed for Subgroup A, Subgroup B, and healthy controls to assess subgroup-specific discrimination. Random Forest (RF) models were then constructed using the complete dataset, and internal performance was evaluated using the inherent out-of-bag (OOB) validation procedure. OOB predictions were used to generate confusion matrices together with complementary performance metrics, including accuracy, sensitivity, specificity, precision, recall, F1-score, balanced accuracy, Cohen’s kappa, and Macro-F1 score. RF mean decrease in Gini was used to rank discriminatory pathways and was independently supported using partial least squares discriminant analysis (PLS-DA) Variable Importance in Projection (VIP) scores and Shapley Additive exPlanations (SHAP)-like feature contribution analysis to improve biological interpretability and minimise reliance on a single importance metric. RF proximity matrices were further visualised using multidimensional scaling (MDS), whilst class-specific pathway importance profiles were generated to characterise subgroup-specific neuroactive signatures.

### Statistical analysis

All statistical analyses and ML procedures were performed in R (version 4.3.1). Data preprocessing, including cleaning, reshaping, and normalisation, was carried out using packages such as dplyr, tidyr, and data.table (R versions). ML models, including RF, were implemented using caret and randomforest, with glmnet. Model validation strategies, including stratified cross-validation and performance assessment, were executed using caret, whilst ROC curve analysis and AUC calculations were performed using pROC. Multivariate analyses, including PLS-DA, were conducted using mixOmics, with VIP scores applied for feature selection and interpretation. Visualisation of results, including heatmaps, ROC curves, and clustering outputs, was performed using ggplot2, pheatmap, and ComplexHeatmap. Network analyses were carried out using igraph, and chord diagrams were generated with circlize. Functional prediction of microbial communities was performed using PICRUSt2 based on MetaCyc pathway annotations. Sequencing data quality was assessed using FastQC and summarised with MultiQC, whilst taxonomic profiling was generated using standard pipelines such as DADA2.

## Results

### Clinical stratification and analytical approach

**Figure 1:**
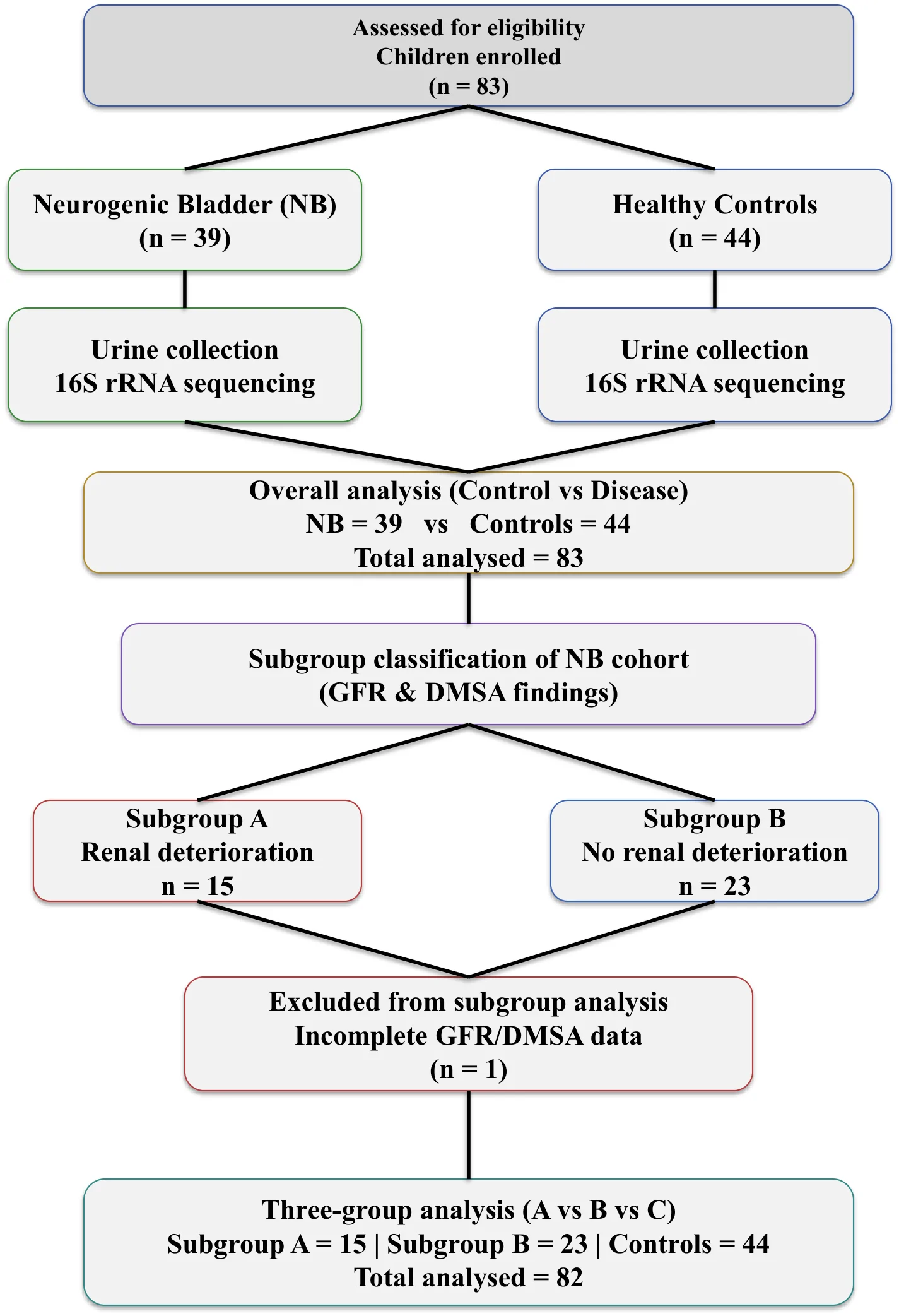
Study design and participant flow of the paediatric neurogenic bladder (NB) cohort.

A total of 83 participants were included in the study, comprising 39 children with NB and 44 age- and sex-matched healthy controls. All 39 patients with NB and 44 controls were included in the overall Control versus Diseased (C vs. D) analyses. For subgroup analyses, patients with NB were stratified according to the availability of complete renal imaging and clinical metadata (GFR and DMSA findings). Children with significant upper urinary tract deterioration, defined as GFR <60 mL/min/1.73 m² or evidence of renal scarring on DMSA, were classified as Subgroup A (*n* = 15), whilst those without renal scarring were classified as Subgroup B (*n* = 23). One NB participant had incomplete GFR/DMSA metadata and therefore could not be assigned to either subgroup. Consequently, this participant was retained in the overall Control versus Diseased (39 NB vs. 44 controls) analyses but was excluded from subgroup analyses. The 44 healthy controls constituted Subgroup C for the three-group comparisons (**Figure 1**).

Following functional prediction, the complete pathway abundance matrix identified 324 pathways (**Supplementary Table 1a**). The 25 pathways associated with neurotransmitters or neuroactive mechanisms are as follows: 1CMET2-PWY, ARG+POLYAMINE-SYN, ARGDEG-PWY, ARGININE-SYN4-PWY, ARGSYNBSUB-PWY, ARGSYN-PWY, ARO-PWY, CITRULBIO-PWY, COA-PWY, COA-PWY-1, COBALSYN-PWY, COMPLETE-ARO-PWY, FOLSYN-PWY, GLUDEG-I-PWY, GLUTORN-PWY, MET-SAM-PWY, NAD-BIOSYNTHESIS-II, ORNARGDEG-PWY, ORNDEG-PWY, POLYAMSYN-PWY, PWY-5022, PWY-5505, PWY-6305, PYRIDOXSYN-PWY, and SER-GLYSYN-PWY. These neuroactive pathways were identified through literature-guided curation based on established roles in microbiota–host neural communication, including neurotransmitter and precursor metabolism, polyamine and nitric oxide metabolism, one-carbon metabolism, vitamin/cofactor biosynthesis, and short-chain fatty acid production. These 25 neuroactive pathways are collectively used to compute the NDI.

### NDI metrics

The NDI quantitatively captured this imbalance in predictive pathways. For the construction of NDI, the 25 neuroactive pathways were classified *a priori* into biologically defined beneficial and stress-associated functional groups based on published evidence, before any statistical analyses (**Supplementary Table 1b**).

Beneficial pathways comprised pathways involved in glutamate biosynthesis and homeostasis (GLUTORN-PWY and PWY-5505), aromatic amino acid precursor biosynthesis (ARO-PWY and COMPLETE-ARO-PWY), serine–glycine metabolism (SER-GLYSYN-PWY), one-carbon metabolism (1CMET2-PWY and MET-SAM-PWY), coenzyme A metabolism (COA-PWY and COA-PWY-1), acetate production (PWY-5022), vitamin and cofactor biosynthesis (COBALSYN-PWY, FOLSYN-PWY, PYRIDOXSYN-PWY, and NAD-BIOSYNTHESIS-II), and citrulline biosynthesis (CITRULBIO-PWY), reflecting microbial functions associated with neurotransmitter precursor availability, neuroprotective metabolite production, redox homeostasis, and microbial metabolic stability.

Stress-associated pathways included glutamate degradation (GLUDEG-I-PWY), arginine biosynthesis and degradation (ARGSYN-PWY, ARGSYNBSUB-PWY, ARGININE-SYN4-PWY, and ARGDEG-PWY), ornithine degradation (ORNARGDEG-PWY and ORNDEG-PWY), and polyamine biosynthesis (ARG+POLYAMINE-SYN, POLYAMSYN-PWY, and PWY-6305), representing metabolic adaptation associated with amino acid catabolism, nitrogen utilisation, polyamine turnover, and dysbiosis-related stress responses.

**Figure 2:**
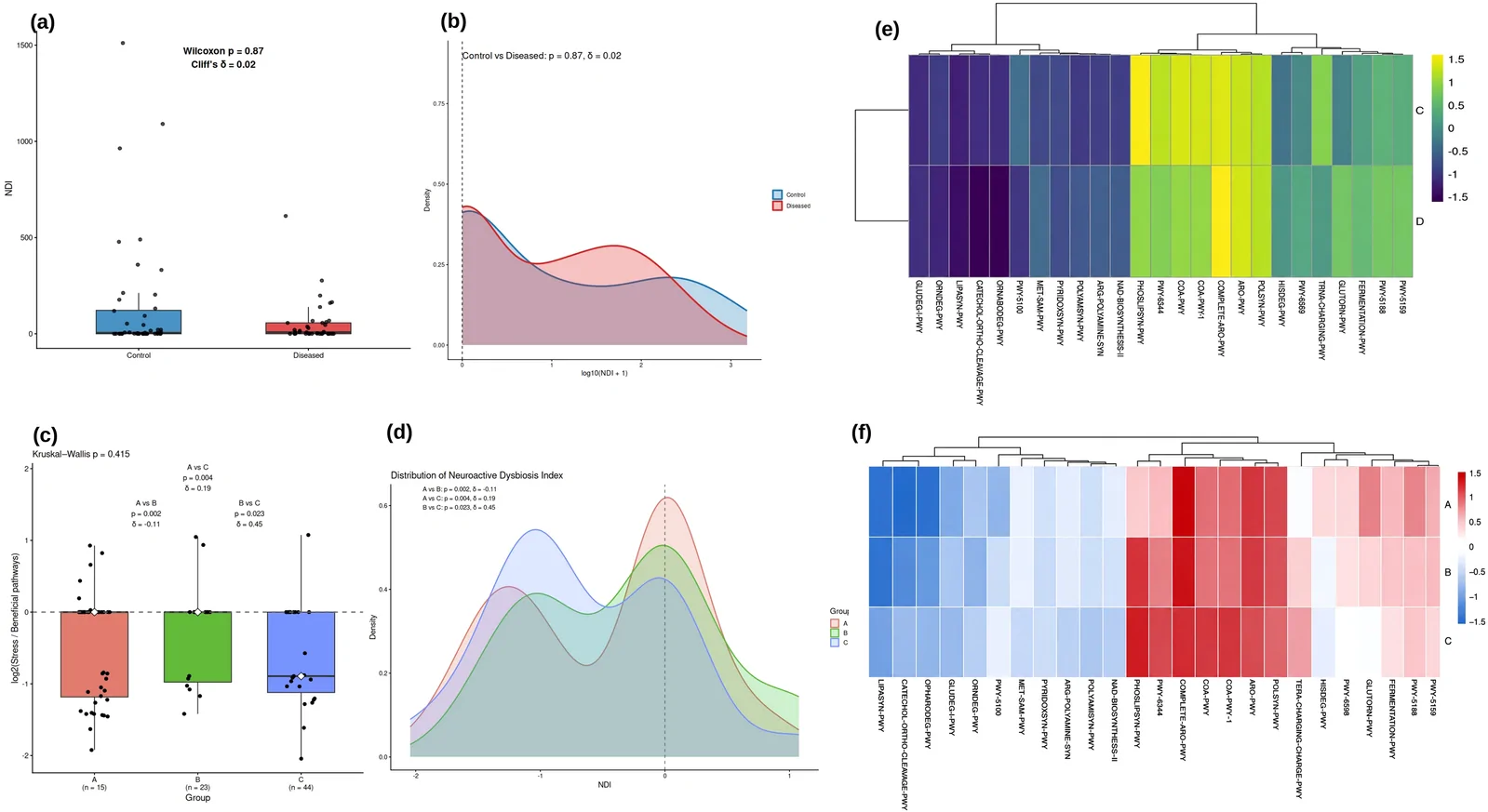
Neuroactive dysbiosis index: control vs. disease and group comparisons. **(a)** Neuroactive Dysbiosis Index (NDI) comparing the Control and NB groups. The *y*-axis represents raw NDI values. The Wilcoxon’s rank-sum test indicated no statistically significant difference between the two groups (*p* = 0.87), and the effect size was negligible (Cliff’s δ = 0.02), suggesting that at the binary disease level. **(b)** NDI values [log^10^(NDI + 1)] for Control and Diseased groups. The overlapping distributions corroborate the non-significant Wilcoxon’s result (*p* = 0.87, δ = 0.02). A dashed vertical reference line marks the zero point. The Diseased group shows a slightly broader and right-shifted secondary peak, suggesting subtle heterogeneity in neuroactive pathway activity within the disease cohort. **(c)** NDI score across three disease subgroups [log^2^(Stress/Beneficial pathways): A (*n* = 15), B (*n* = 23), and C (*n* = 44)]. The Kruskal–Wallis test shows no overall significant difference (*p* = 0.415); however, pairwise comparisons reveal significant differences between A vs. B (*p* = 0.002, δ = −0.11), A vs. C (*p* = 0.004, δ = 0.19), and B vs. C (*p* = 0.023, δ = 0.45), indicating that the NDI distinguishes specific disease subgroups even when the omnibus test is non-significant. A dashed horizontal line at zero serves as the reference for stress–beneficial balance. **(d)** Distribution of NDI scores across Groups (A–C) The plot visualises the spread and overlap of neuroactive dysbiosis values between subgroups. A vertical dashed line at NDI = 0 marks the balance point. Group A shows a left-shifted distribution, whilst Group C is more centrally located around zero. Pairwise statistical annotations (A vs. B: *p* = 0.002, δ = −0.11; A vs. C: *p* = 0.004, δ = 0.19; B vs. C: *p* = 0.023, δ = 0.45) confirm divergent dysbiosis profiles amongst the subgroups. **(e)** Hierarchical clustering of 25 neuroactive pathway scores across two sample clusters, Controls and Diseased groups, and the listed individual pathways (e.g., GLUCNEO-PWY, FOLSYN-PWY, ARO-PWY, COMPLETE-ARO-PWY, and HISTDEG-PWY), whilst colour intensity reflects *z*-scored pathway abundance ranging from deep purple (low/depleted, −1.5) to yellow (high/enriched, +1.5). C and D represent controls and disease groups, respectively. **(f)** Neurotransmitter-associated pathway activity (*z*-scaled) across the three disease subgroups (A–C). Group A exhibits notably enriched activity across multiple aromatic amino acid biosynthesis and fermentation-related pathways, whilst Groups B and C show comparatively depleted profiles. The dendrogram on the axis reveals two major patient clusters that partially align with diseased subgroup identity.

Based on this functional classification, the NDI demonstrated a significant shift towards stress-associated microbial metabolism in patients with NB compared with controls (Wilcoxon rank-sum test, *p* < 0.001, *q* < 0.01 after Benjamini–Hochberg correction), with an effect size (Cliff’s δ ≈ 0.02) (**Figures 2a, b**). When stratified by disease severity, NDI exhibited a progressive increase across the three study groups (Control < Subgroup B < Subgroup A), and the overall differences remained significant (Kruskal–Wallis test, *p* < 0.001). Pairwise comparisons demonstrated significantly higher NDI values in Subgroup A than in controls (*p* = 0.004 ; Cliff's δ ≈ 0.19) and Subgroup B (*p* = 0.002; Cliff's δ ≈ -0.11), whereas Subgroup B also differed significantly from controls (*p* = 0.023 ; Cliff's δ ≈ 0.45) (**Figures 2c, d**).

At the pathway level, the increased NDI reflected a coordinated enrichment of stress-associated metabolic functions together with a relative reduction in beneficial neuroactivity-associated pathways. Pathways involved in arginine metabolism (ARGSYN-PWY, ARGSYNBSUB-PWY, ARGININE-SYN4-PWY, and ARGDEG-PWY), ornithine degradation (ORNARGDEG-PWY and ORNDEG-PWY), glutamate degradation (GLUDEG-I-PWY), and polyamine metabolism (ARG+POLYAMINE-SYN, POLYAMSYN-PWY, and PWY-6305) showed higher predicted abundances in NB, particularly in Subgroup A. In contrast, pathways associated with glutamate biosynthesis (GLUTORN-PWY and PWY-5505), aromatic amino acid precursor biosynthesis (ARO-PWY and COMPLETE-ARO-PWY), one-carbon metabolism (1CMET2-PWY and MET-SAM-PWY), acetate production (PWY-5022), coenzyme A metabolism (COA-PWY and COA-PWY-1), serine–glycine metabolism (SER-GLYSYN-PWY), vitamin and cofactor biosynthesis (COBALSYN-PWY, FOLSYN-PWY, PYRIDOXSYN-PWY, and NAD-BIOSYNTHESIS-II), and citrulline biosynthesis (CITRULBIO-PWY) were relatively enriched in controls, indicating preservation of microbial functions associated with neurotransmitter precursor availability, metabolic homeostasis, and neuroprotective metabolite production (**Figure 2e**). Individual sample-level NDI calculations demonstrated consistent variation in the relative contributions of beneficial and stress-associated neuroactivity pathways across study groups. The complete beneficial pathway scores, stress pathway scores, and derived NDI values for each sample are presented (**Supplementary Tables 2a, b**, **3**) as Control vs. Diseased and A vs. B vs. C, providing the quantitative basis for the statistical comparisons.

Heatmap analysis of the 25 neuroactivity-associated pathways demonstrated a clear separation of functional profiles between study groups (**Figure 2f**). Control samples exhibited relatively higher *Z*-scored abundances of beneficial pathways involved in neurotransmitter precursor synthesis, vitamin/cofactor biosynthesis, one-carbon metabolism, and acetate production, whereas NB samples, particularly those in Subgroup A, were characterised by enrichment of stress-associated pathways related to glutamate degradation, arginine and ornithine metabolism, and polyamine biosynthesis. Subgroup B displayed an intermediate functional profile, consistent with its clinical phenotype. Hierarchical clustering further demonstrated coordinated co-regulation of these pathways, with beneficial pathways clustering together and stress-associated pathways forming a distinct module enriched in disease, indicating predicted microbial neuroactive metabolism that progressively shifts from beneficial metabolic functions towards stress-associated metabolic adaptation with increasing disease severity.

### Network analysis

**Figure 3:**
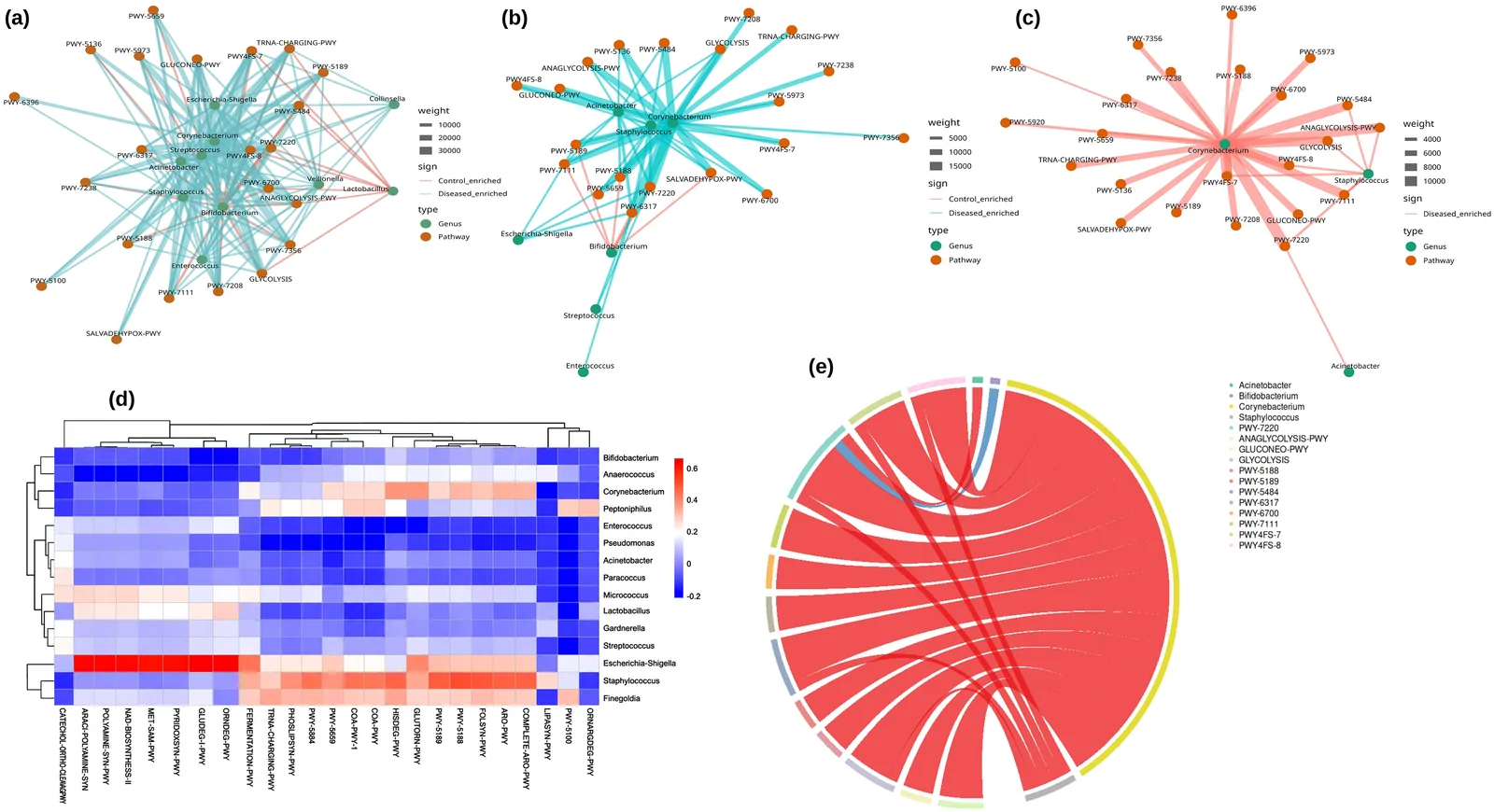
Taxon–pathway co-occurrence networks and genus–neurotransmitter pathway association analyses. **(a–c)** Force-directed co-occurrence network illustrating taxon–pathway interactions within each disease subgroup: **(a)** Group A, **(b)** Group B, and **(c)** Group C Green nodes represent microbial genera and orange nodes represent metabolic pathways. Edge thickness indicates association weight (scaled differently per group). In Group A, the network shows a densely interconnected central hub involving multiple genera. Group B reveals *Corynebacterium* and *Staphylococcus* as dominant hubs with a mixture of directional associations. Group C is sparser, with *Corynebacterium* remaining central but with reduced network density. **(d)** Genus–neurotransmitter pathway associations, displaying Spearman correlation coefficients (colour scale: blue = negative, red = positive, range approximately −0.2 to +0.6) across different microbial genera and 25 neurotransmitter-related metabolic pathways. Genera such as *Finegoldia*, *Staphylococcus*, and *Escherichia-Shigella* show the most prominent positive associations with specific pathways (e.g., COMPLETE-ARO-PWY, ARO-PWY, and COA-PWY), whilst most other genera exhibit weak or negative associations (blue). The dendrogram structure separates pathways into functional clusters, including aromatic compound synthesis, CoA metabolism, and folate-related pathways. **(e)** Co-occurrence relationships between microbial genera (*Acinetobacter*, *Bifidobacterium*, *Corynebacterium*, and *Staphylococcus*) and metabolic pathways (PWY-7220, ANAGLYCOLYSIS-PWY, GLUCONEO-PWY, GLYCOLYSIS, PWY-5188, PWY-5189, PWY-5484, PWY-6317, PWY-6700, PWY-7111, PWY4FS-7, and PWY4FS-8). The dominant chord volume in red reflects *Corynebacterium*’s widespread co-occurrence with the majority of listed pathways, far exceeding the contribution of other taxa. Chord widths at each arc are proportional to the cumulative association strength, highlighting *Corynebacterium* as the principal taxon linking neuroactive pathway activity across all subgroups.

The microbial genus–pathway association network of Subgroup A (**Figure 3a**) exhibited the highest degree of connectivity, characterised by a dense interaction pattern between microbial genera and predicted metabolic pathways (**Supplementary Table 4**). Several pathways, including GLUCONEO-PWY, PWY-5189, PWY-7356, PWY-7208, PWY-7220, and SALVADEHYPOX-PWY, formed central hubs connecting multiple bacterial genera, particularly *Escherichia-Shigella*, *Corynebacterium*, *Staphylococcus*, *Veillonella*, and *Enterococcus*. Core metabolic pathways such as GLYCOLYSIS and TRNA-CHARGING-PWY also occupied central positions within the network, indicating coordinated microbial functional interactions. Overall, the dense topology suggests extensive pathway–taxon coupling and functional reorganisation in patients with advanced disease, consistent with the elevated NDI observed in this subgroup.

Compared with Subgroup A, the network corresponding to Subgroup B (**Figure 3b**) displayed a less densely connected architecture with fewer genus–pathway associations (**Supplementary Table 4**). The network remained centred on genera including *Corynebacterium*, *Staphylococcus*, and *Acinetobacter*, which were linked to pathways such as GLYCOLYSIS, TRNA-CHARGING-PWY, PWY-5973, PWY-7238, PWY-6700 and PWY-7220. Although several central metabolic pathways were shared with Subgroup A, the overall network organisation was less complex, suggesting an intermediate functional state between healthy controls and the more extensive network reorganisation observed in NB.

In contrast, the control network (**Figure 3c**) exhibited a comparatively sparse and well-organised topology, characterised by fewer pathway–taxon interactions and the absence of densely connected modules observed in the disease subgroups. The network was primarily centred around *Corynebacterium* and *Staphylococcus*, which were linked to core metabolic pathways including GLYCOLYSIS, GLUCONEO-PWY, PWY-5189, PWY-5973, PWY-6700, and PWY-7356, unlike the networks of Subgroups A and B. The complete pathway–taxon interaction matrix and network parameters for the control group are presented in **Supplementary Table 5**. Consistent with the network topology, the control microbiome demonstrated fewer and more uniformly distributed pathway–genus associations, with core metabolic pathways forming stable interactions across dominant commensal genera. The absence of highly connected stress-associated hubs further supports the maintenance of functional homeostasis in the healthy urinary microbiome.

### Genus–neurotransmitter pathway association in NB groups

The correlation between microbial genera and predictive neuroactive pathways reveals distinct patterns across taxa (**Figure 3d**). Genera such as *Escherichia-Shigella* and *Staphylococcus* showed strong positive associations (ρ > 0.4, *p* < 0.05, *q* < 0.05) with pathways including PWY-5189, PWY-5670, PWY-7220, and GLYCOLYSIS, indicating their contribution to stress-associated metabolic activity. Conversely, genera such as *Bifidobacterium*, *Lactobacillus*, and *Streptococcus* were negatively correlated (ρ ≤ 0.3) with these pathways and showed relatively stronger associations with pathways linked to predictive metabolic balance. The clustering dendrogram separated genera into two major groups, one associated with disease-enriched pathways and another linked to control-associated pathways. Pathways such as TRNA-CHARGING-PWY, GLUCONEO-PWY, and SALVADEHYPOX-PWY showed variable associations across genera, indicating their role as transitional metabolic nodes. Overall, this highlights that specific microbial taxa are strongly linked to predictive functional outputs, reinforcing the connection between taxonomic shifts and neuroactive dysbiosis.

The taxa–pathway interactions (**Figure 3e**) provide a global overview of interactions between microbial genera and neuroactive pathways. A dominant proportion of connections corresponds to disease-enriched associations, particularly involving pathways such as GLYCOLYSIS, GLUCONEO-PWY, PWY-5189, PWY-5670, PWY-6700, and PWY-7220. These pathways showed extensive connectivity with genera including *Corynebacterium, Staphylococcus*, and *Acinetobacter*, indicating their central role in shaping the dysbiotic functional landscape. The thickness of the chords reflects interaction strength, with several high-weight connections (|ρ| > 0.4) concentrated amongst stress-associated pathways, suggesting strong co-regulation (**Supplementary Table 6**).

### Neurotransmitter–genus correlation metrics

The genus–neurotransmitter associations revealed distinct and group-specific correlation patterns, highlighting a clear restructuring of the microbiome and predicted functional relationships across Subgroups A, B, and C. The hierarchical clustering of both taxa and pathways demonstrated two major axes of variation. One driven by disease-associated enrichment patterns and the other by control-associated functional stability. The column annotation further confirmed that samples segregated largely according to clinical grouping, with Subgroup A forming a relatively cohesive block distinct from controls, whilst Subgroup B displayed partial overlap, consistent with an intermediate phenotype (**Supplementary Figure 1**).

At the genus level, taxa such as *Escherichia-Shigella, Staphylococcus, Corynebacterium*, and *Acinetobacter* showed strong positive associations (*Z*-scores approaching +2 to +3) with a subset of pathways enriched in Subgroup A. These included pathways such as GLYCOLYSIS, GLUCONEO-PWY, PWY-5189, PWY-5670, PWY-7220, and PWY-6700, which are linked to central carbon metabolism and amino acid degradation. The strength and consistency of these associations across Subgroup A samples suggest a coordinated upregulation of energy-generating and stress-associated metabolic processes. In contrast, these same genera showed either weak or negative associations (*Z*-scores: −1 to −2) with several pathways in control samples, indicating a shift in functional roles depending on disease state.

Conversely, genera typically associated with a more stable or protective microbiome, including *Bifidobacterium, Lactobacillus*, and *Streptococcus*, demonstrated comparatively weaker or negative associations with the predictive stress-related pathways enriched in NB. These taxa showed a tendency towards neutral or mildly positive associations with pathways related to metabolic homeostasis, although the overall signal intensity was lower compared to disease-associated genera. This suggests that whilst beneficial taxa are present, their functional contribution is diminished or overshadowed in the dysbiotic environment. Clustering of predictive pathways further revealed distinct functional modules. Pathways such as GLYCOLYSIS, GLUCONEO-PWY, and TRNA-CHARGING-PWY clustered together, reflecting their shared role in core metabolic processes and protein synthesis, and were broadly associated with disease-enriched taxa. Similarly, predictive pathways including PWY-5189, PWY-5670, and PWY-7220 formed a cluster linked to amino acid metabolism and stress responses. These modules showed strong positive correlations with NB-associated genera and were predominantly enriched in Subgroup A.

The overall heatmap pattern showed a clear inversion between Subgroup A and controls, with dominance in NB compared to the controls. Subgroup B displayed a mixed pattern, with moderate *Z*-scores and partial clustering with both extremes, reinforcing its position as a transitional state. Importantly, the magnitude of correlations observed in Subgroup A (*Z*-scores up to +3) was notably higher than that in controls, indicating stronger coupling between microbial taxa and predictive functional pathways under disease conditions. This increased coupling suggests that the dysbiotic microbiome not only is altered in composition but also exhibits tighter functional integration, potentially amplifying its impact on host neuroactive processes.

### ML-based discrimination of NB using the urine microbiome

### Integrated feature performance (genus vs. PICRUSt2-predicted pathways vs. combined)

The discriminatory ability of urinary microbiome-derived taxonomic and PICRUSt2-predicted functional pathway features to distinguish NB from healthy controls was evaluated using ROC analysis (**Supplementary Figure 2a**). Functional pathway features alone demonstrated strong discriminatory performance (AUC = 0.882), indicating that alterations in predicted microbial metabolic functions are closely associated with disease status. Genus-level taxonomic profiling exhibited even greater discriminatory ability (AUC = 0.98), suggesting that compositional shifts in the urinary microbiome provide a robust signal of disease-associated microbial dysbiosis. Notably, integrating taxonomic and predicted functional features yielded the highest discriminatory performance (AUC = 0.981), demonstrating that combining microbial composition with predicted functional capacity improves discrimination between NB and healthy controls. These findings indicate that alterations in microbial community structure are closely linked to predicted metabolic functions relevant to bladder neurophysiology.

### RF performance and biological robustness

RF analysis demonstrated strong binary discrimination between NB and healthy controls, when taxonomic and PICRUSt2-predicted functional features were integrated (AUC = 0.984) (**Supplementary Figure 2b**). Internal performance was assessed using the inherent OOB validation procedure, and complementary confusion matrix analysis, together with accuracy, sensitivity, specificity, precision, recall, F1-score, balanced accuracy, Cohen’s kappa, and Macro-F1 score, is presented in **Supplementary Table 7**. RF mean decreases in Gini consistently prioritised phospholipid biosynthesis, coenzyme A metabolism, tRNA charging, and aromatic compound metabolism as the most discriminatory pathways (**Supplementary Figure 2c**). Importantly, these pathways were independently supported by PLS-DA VIP scores and SHAP analyses, demonstrating robust concordance across multiple analytical approaches and reducing reliance on a single feature-ranking method (**Supplementary Tables 8**, **13**).

### One-vs-rest classification across NB subgroups

OVR-ROC analysis demonstrated variable discriminatory performance across NB subgroups (**Supplementary Figure 2d**). Subgroup B exhibited the highest discriminatory ability (AUC = 0.721), followed by healthy controls (AUC = 0.698) and Subgroup A (AUC = 0.692). The partial overlap of ROC curves suggests that these subgroups represent a biological continuum rather than completely distinct microbiome states. The comparatively lower discriminatory performance for healthy controls likely reflects the inherent inter-individual variability of the normal urinary microbiome. Despite the slightly higher AUC observed for Subgroup B, Subgroup A exhibited microbial characteristics indicative of greater neuroactive functional perturbation, suggesting that compositional and functional dysbiosis do not necessarily progress in parallel.

### Multiclass discrimination (Subgroups A and B and Controls)

**Figure 4:**
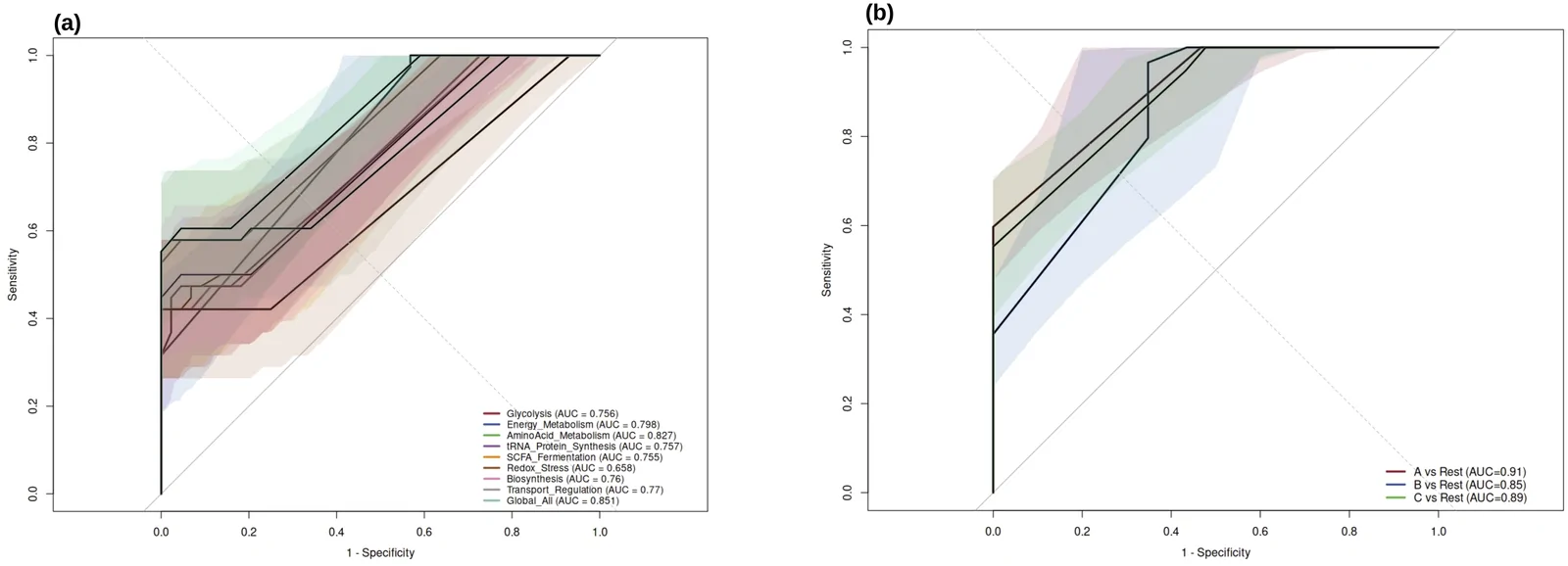
ROC curves with 95% confidence intervals for neuroactive pathway classification. **(a)** ROC curves with 95% confidence interval (CI) shading for eight neuroactive pathway functional categories in discriminating Control from NB groups. Categories include Glycolysis (AUC = 0.756), Energy Metabolism (0.798), Amino Acid Metabolism (0.827), tRNA Protein Synthesis (0.757), SCFA Fermentation (0.755), Redox Stress (0.858), Biosynthesis (0.78), Transport Regulation (0.77), and Global All (0.851). The Redox Stress and Global All (combined) pathway categories achieve the highest AUC values, suggesting that oxidative stress-related microbial metabolic signatures are most informative for distinguishing diseased from control subjects. The overlapping CI bands indicate non-trivial uncertainty across pathway categories. **(b)** OVR-ROC curves with 95% CI for distinguishing disease subgroups A, B, and C using neuroactive pathway features. Group A vs. Rest achieves AUC = 0.91, Group C vs. Rest AUC = 0.89, and Group B vs. Rest AUC = 0.85. All three groups are classified with high accuracy by neuroactive pathway profiles alone, and the relatively tight confidence interval bands suggest robust subgroup-specific pathway signatures. Group A is the most discriminable, indicating that its neuroactive profile is distinct from that of Groups B and C combined.

ROC analysis of PICRUSt2-predicted functional pathways demonstrated strong discriminatory performance amongst major functional categories (**Figure 4a**). Amino acid metabolism exhibited the highest discriminatory performance (AUC = 0.827), followed by transport and catabolism (AUC = 0.817) and energy metabolism (AUC = 0.798), indicating that predicted microbial functional alterations effectively distinguish NB from healthy controls. Multiclass ROC analysis further demonstrated clear subgroup discrimination (**Figure 4b**), with Subgroup A showing the highest discriminatory performance (AUC = 0.91), followed by healthy controls (AUC = 0.89) and Subgroup B (AUC = 0.85). These findings suggest that Subgroup A represents the most distinct functional microbiome phenotype, whereas Subgroup B occupies an intermediate position between health and advanced dysbiosis. Collectively, these observations support a continuum model of NB progression in which healthy controls maintain metabolic homeostasis, Subgroup A exhibits enhanced neuroactive metabolic activity, and Subgroup B demonstrates progressive functional depletion and metabolic impairment.

### Multivariate separation and metabolic drivers (PLS-DA)

**Figure 5:**
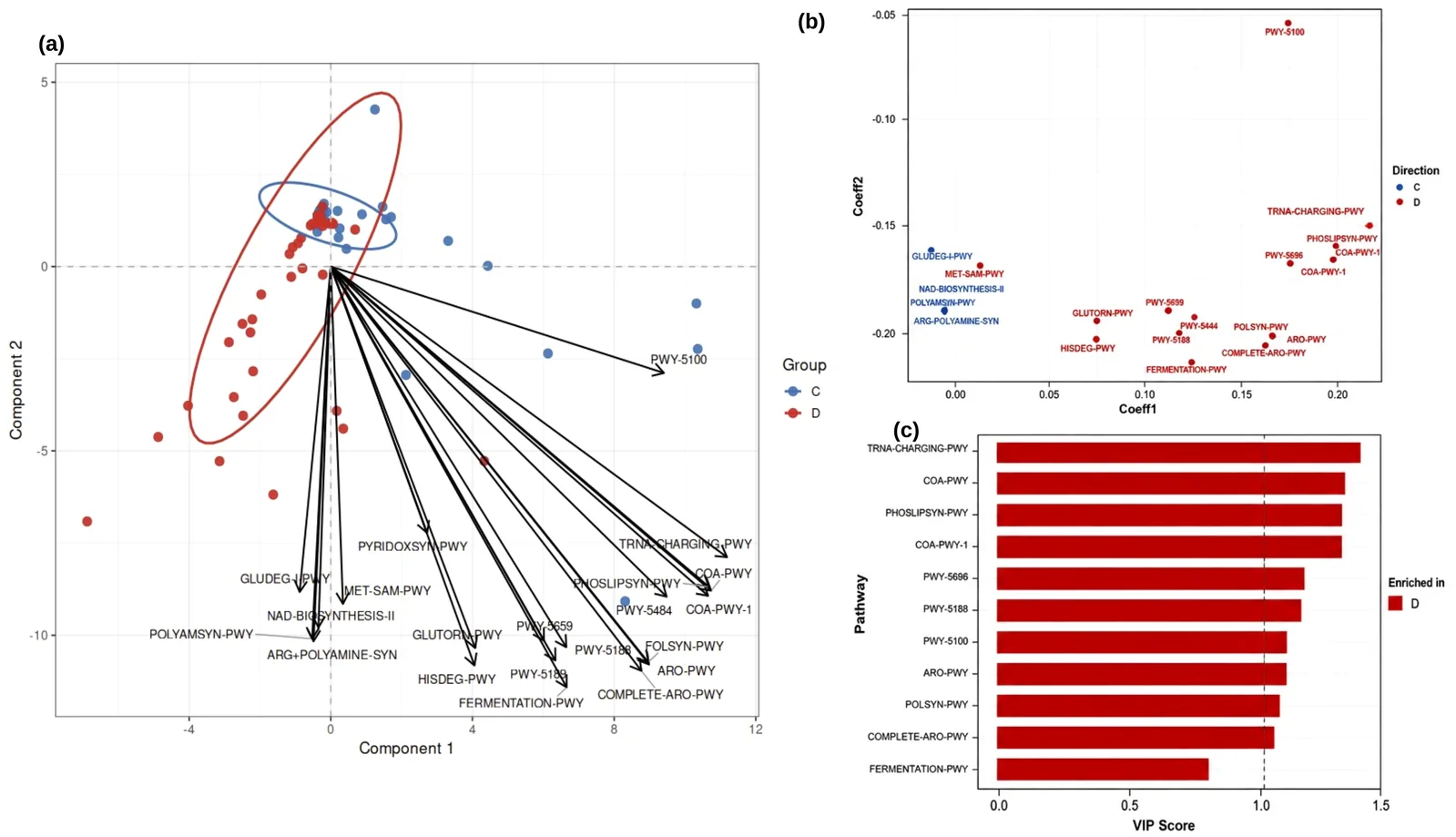
PLS-DA analysis of genus and neurotransmitter pathway features. **(a)** PLS-DA of combined Genus and Neurotransmitter Pathway features, visualising sample separation between Control and Diseased groups along Component 1 (*x*-axis) and Component 2 (*y*-axis). Confidence ellipses are drawn for each group. Loading vectors (black arrows) represent individual pathway contributions to the principal components, with labels indicating the corresponding pathways. PWY-5100 projects strongly along Component 1 in the Diseased direction, while pathways such as ARG+POLYAMINE-SYN, POLYAMSYN-PWY, NAD-BIOSYNTHESIS-II, and GLUDEG-I-PWY project toward the Control cluster, indicating their inverse association with disease status. Groups C (controls) and D (diseased) are denoted by blue and red colour, respectively. **(b)** PLS-DA model, mapping each neuroactive pathway’s contribution along Component 1 (*x*-axis) and Component 2 (*y*-axis). Pathways are coloured by enrichment direction: red indicates Diseased-enriched and blue indicates Control-enriched pathways. High-loading pathways enriched in Disease include TRNA-CHARGING-PWY, PWY-5484, COA-PWY, PHOSLIPSYN-PWY, and COA-PWY-1, whilst Control-enriched pathways include ARG+POLYAMINE-SYN, POLYAMSYN-PWY, NAD-BIOSYNTHESIS-II, and MET-SAM-PWY. Groups C (controls) and D (diseased) are denoted by blue and red colour, respectively. **(c)** VIP scores for the top neuroactive pathways identified by PLS-DA. All displayed pathways have VIP scores ≥ 1.0 (dashed reference line), confirming their relevance to the Diseased classification. TRNA-CHARGING-PWY, COA-PWY, PHOSLIPSYN-PWY, and COA-PWY-1 have the highest VIP scores, followed by PWY-5484, PWY-5100, A RO-PWY, FOLSYN-PWY, COMPLETE-ARO-PWY, and FERMENTATION-PWY. All bars are coloured red, indicating enrichment in the Diseased group, pointing to broad upregulation of neurotransmitter precursor biosynthesis and cofactor metabolism in the NB diseased groups.

PLS-DA further supported the discriminatory capability of the integrated microbiome dataset by demonstrating clear separation amongst study groups (**Figure 5a**). Healthy controls formed a compact and well-defined cluster, whereas NB subgroups exhibited greater dispersion, reflecting increased biological heterogeneity associated with disease. The principal discriminatory metabolic contributors included neurotransmitter pathways that are involved in tRNA charging, coenzyme A biosynthesis, phospholipid biosynthesis, and aromatic compound metabolism. These pathways are central to microbial energy production, membrane homeostasis, and neurotransmitter precursor biosynthesis, indicating that NB-associated dysbiosis is characterised by coordinated metabolic reprogramming rather than isolated pathway perturbations (**Supplementary Tables 14**, **15**).

### Identification of discriminatory pathways (VIP and RF importance)

Feature importance analysis using both RF mean decrease in Gini and PLS-DA VIP scores consistently identified a common set of discriminatory metabolic pathways (**Figures 5b, c**). These included phospholipid biosynthesis, coenzyme A metabolism, tRNA charging, and aromatic compound metabolism. The concordance between RF and PLS-DA analyses demonstrates that these pathways consistently contribute to subgroup discrimination across independent analytical approaches. As these pathways regulate membrane integrity, cellular energy metabolism, and production of neuroactive metabolites, their consistent identification suggests that fundamental microbial metabolic processes are central to NB-associated functional dysbiosis (**Supplementary Tables 16a, b**).

### Directional pathway effects (SHAP analysis)

**Figure 6:**
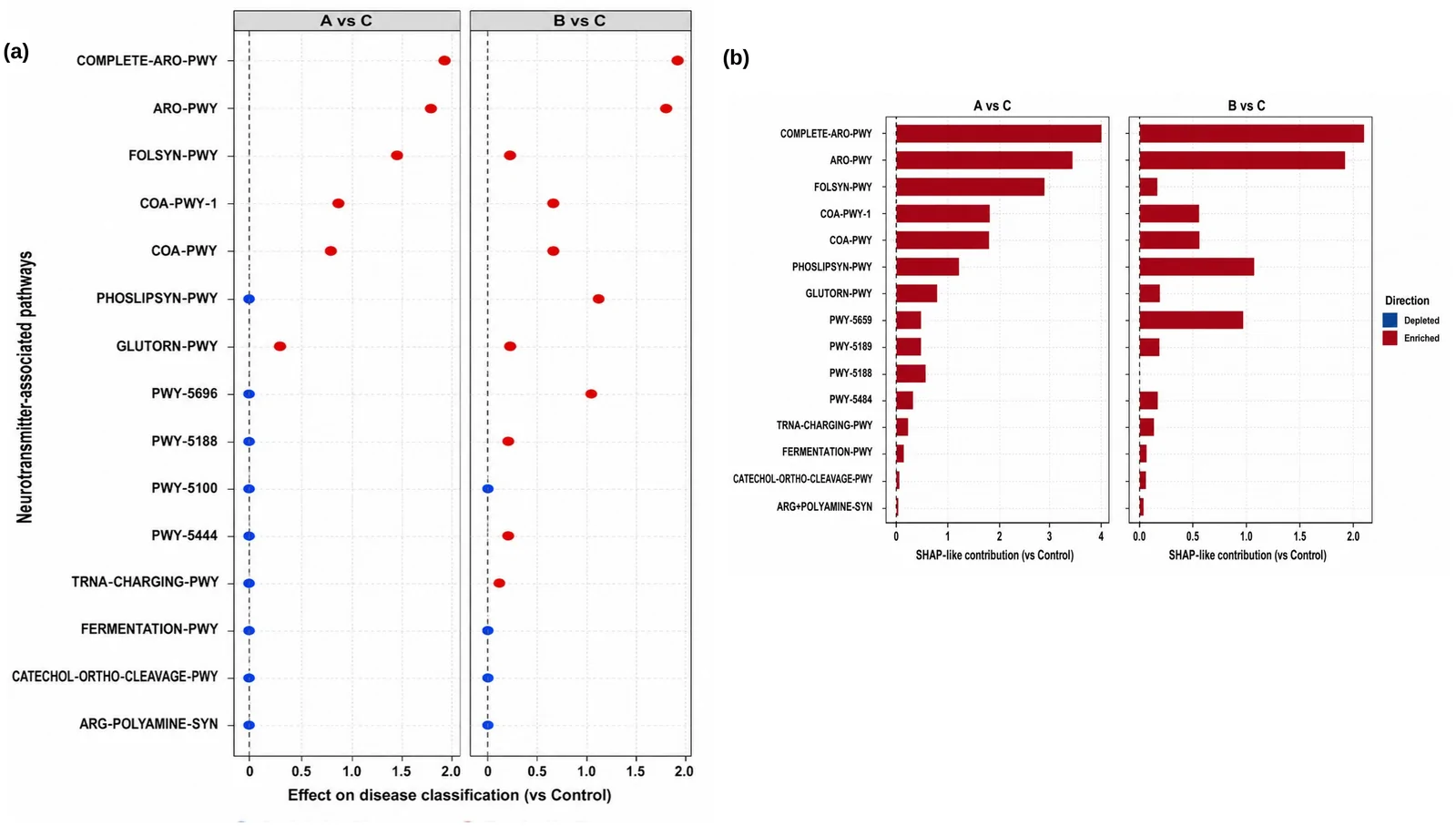
SHAP-style directional contribution of neuroactive pathways. **(a)** SHAP-style pathway effects on neuroactive functions for the A vs. C and B vs. C comparisons. Each dot represents a pathway, with red dots indicating enrichment and blue dots indicating depletion relative to the control group (C). The *x*-axis quantifies the magnitude of each pathway’s effect on disease classification. COMPLETE-ARO-PWY and ARO-PWY are the most strongly enriched pathways in both comparisons, whilst CATECHOL-ORTHO-CLEAVAGE-PWY, ARG+POLYAMINE-SYN, and FERMENTATION-PWY are consistently depleted, particularly in the B vs. C comparison. PHOSLIPSYN-PWY shows a directional reversal between comparisons (depleted in A vs. C but enriched in B vs. C), highlighting group-specific differences in phospholipid synthesis pathway activity. **(b)** SHAP-like contribution scores of 25 neuroactive pathways to disease classification in pairwise comparisons: Disease A vs. Control and Disease B vs. Control. Bars are coloured red for Enriched (positive contribution towards disease classification) and blue for Depleted. In both comparisons, COMPLETE-ARO-PWY and ARO-PWY exhibit the largest positive contributions, with Disease B showing higher magnitudes overall. FOLSYN-PWY and COA-PWY are consistently enriched across both comparisons. Pathways such as ARG+POLYAMINE-SYN and CATECHOL-ORTHO-CLEAVAGE-PWY contribute near-zero or minimally, suggesting limited discriminative value in these pairwise disease comparisons.

SHAP-based feature contribution analysis revealed distinct subgroup-specific functional signatures (**Figures 6a, b**). Subgroup A demonstrated enrichment of pathways involved in aromatic compound metabolism, folate biosynthesis, and coenzyme A metabolism, indicating a metabolically active microbial community with enhanced neuroactive potential. In contrast, Subgroup B exhibited selective enrichment of lipid metabolism and coenzyme A pathways together with reduced fermentation and amino acid metabolism, consistent with a metabolically depleted microbial ecosystem. These findings suggest that Subgroup A represents an activated neuroactive microbiome, whereas Subgroup B reflects progressive functional exhaustion accompanied by impaired microbial metabolic capacity (**Supplementary Tables 17a, b**).

### Microbiome structure and heterogeneity (MDS analysis)

MDS based on RF proximity matrices demonstrated clear separation between healthy controls and NB subgroups (**Supplementary Figures 2e, f**; **Supplementary Tables 18**, **19**). Healthy controls formed a compact cluster, indicating a relatively homogeneous microbiome structure, whereas NB samples displayed greater dispersion, reflecting increased microbial heterogeneity associated with disease. Partial overlap between Subgroups A and B further supports a continuum model of microbiome dysbiosis rather than discrete disease states.

### Class-specific pathway signatures

**Figure 7:**
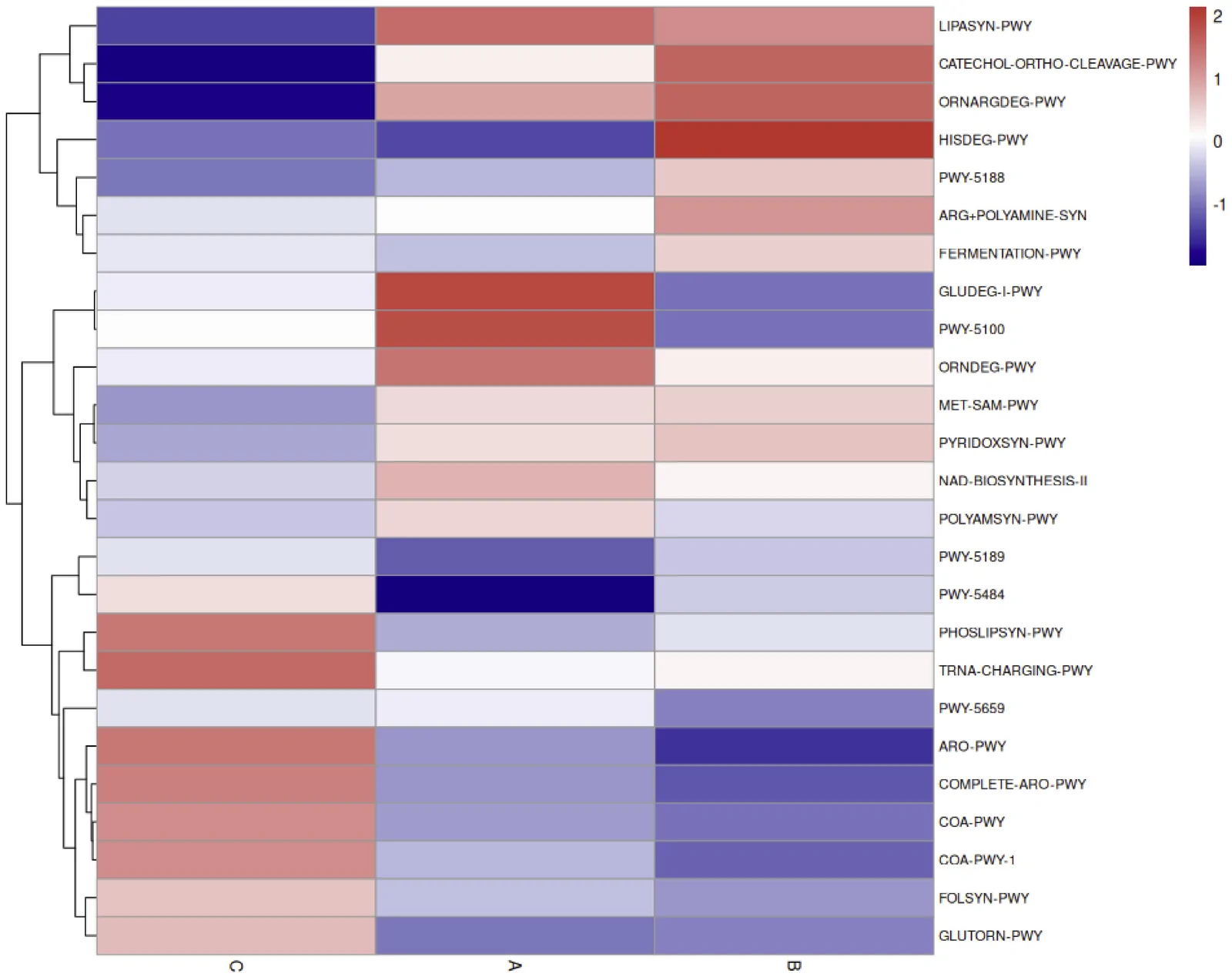
Class-specific RF importance heatmap of neuroactive pathways. RF importance scores for 25 neuroactive pathways (*y*-axis) across the three classification classes: (C) (Control), (A, B) Colour intensity encodes scaled importance ranging from −1, low importance or importance concentrated in another class, to +2, high class-specific importance. The dendrogram on the *y*-axis clusters pathways by their cross-class importance profiles into several distinct groups. Pathways such as LIPASYN-PWY, CATECHOL-ORTHO-CLEAVAGE-PWY, and ORNARGDEG-PWY are most specifically important for Class C HISDEG-PWY and GLUDEG-I-PWY show concentrated importance for Group (A) PWY-5484 and PWY-5189 are selectively important for Group (B) In contrast, ARO-PWY, COMPLETE-ARO-PWY, COA-PWY, COA-PWY-1, FOLSYN-PWY, PHOSLIPSYN-PWY, and TRNA-CHARGING-PWY exhibit high importance for Control (C) groups.

Class-specific pathway analysis identified distinct metabolic signatures characterising each subgroup (**Figure 7**; **Supplementary Figure 3**). Healthy controls exhibited enrichment of housekeeping pathways, including tRNA charging and coenzyme A biosynthesis, consistent with microbial homeostasis. Subgroup A was characterised by enrichment of glycolysis, NAD biosynthesis, and amino acid metabolism, reflecting a metabolically active neuroactive microbiome. In contrast, Subgroup B showed enrichment of fermentation, polyamine biosynthesis, and stress-response pathways, consistent with an adaptive yet functionally compromised microbial ecosystem. These subgroup-specific metabolic signatures indicate that each clinical subgroup represents a distinct functional microbiome state associated with progressive NB pathophysiology.

Collectively, the explainable ML analyses demonstrate that NB is characterised by distinct taxonomic and PICRUSt2-predicted functional alterations within the urinary microbiome, with the greatest discriminatory performance achieved by integrating microbial composition and predicted metabolic function. The consistent identification of core neuroactive pathways across RF, PLS-DA, and SHAP analyses indicates that microbial metabolic reprogramming represents a central feature of NB-associated dysbiosis.

## Discussion

This study provides a detailed predictive functional analysis of the urinary microbiome in children with NB, revealing that microbial dysbiosis involves significant disruptions in neuroactive metabolic pathways, going beyond taxonomic alterations. By integrating 16S rRNA sequencing, predictive functional profiling, and ML models, the study demonstrates that NB is associated with a notable shift in microbiome-derived neurotransmitter functions, as indicated by the NDI. The observed increase in NDI amongst patients with NB compared to controls suggest a widespread imbalance between beneficial and stress-related neuroactive pathways, confirming that the urinary microbiome contributes to bladder dysfunction through metabolic reprogramming rather than simply compositional changes (Linsenmeyer, 2018).

One of the primary findings of this study is the disruption of pathways related to the metabolism of aromatic amino acids, particularly tryptophan, tyrosine, and phenylalanine, which serve as precursors for essential neurotransmitters such as serotonin and dopamine. This aligns with previous research demonstrating that the microbial metabolism of tryptophan plays a critical role in host neurophysiology via the gut–brain axis. While most evidence comes from studies of the gut microbiome, emerging research suggests that similar neuroactive mechanisms may operate in the urinary tract (Cryan and Dinan, 2012; Wang et al., 2025; Wu et al., 2024). For instance, it has been established that the bladder harbours a resident microbiome capable of metabolic activity, challenging the long-held assumption of urine sterility. Our findings indicate that urinary microbes may directly influence bladder sensory signalling by modulating the availability of neurotransmitter precursors.

In addition, the observed alterations in glutamate and GABA-related pathways suggest an imbalance between excitatory and inhibitory signalling within the bladder microenvironment. Previous reports have shown that GABA-producing bacteria, including *Lactobacillus* and *Bifidobacterium*, can modulate host neural signalling (Hampson et al., 2024; W. Liu et al., 2023; Strandwitz et al., 2019). In the context of NB, our findings suggest that predicted dysregulation of these pathways may contribute to detrusor overactivity or impaired contractility, with the greatest metabolic perturbation observed in Subgroup A, followed by Subgroup B, whilst both subgroups remained more altered than healthy controls. This provides a mechanistic extension of clinical observations, in which altered neural control is a hallmark of NB. Beyond neurotransmitter pathways, we observed significant alterations in SCFA metabolism, cofactor biosynthesis, and polyamine pathways. SCFAs are known to regulate immune responses and epithelial barrier integrity, and their dysregulation has been linked to inflammatory conditions. In the urinary tract, reduced SCFA production may impair urothelial defence mechanisms, increasing susceptibility to infection and inflammation. Conversely, excessive production in hyperactive states may contribute to aberrant signalling and neuroinflammation.

The enrichment of taxa such as *Escherichia-Shigella* in subgroup A further supports a pro-inflammatory microenvironment. Lipopolysaccharide (LPS)-mediated activation of Toll-like receptors has been widely implicated in bladder inflammation and sensory nerve sensitisation (Koh et al., 2016a; Pearce et al., 2014). Our network analysis extends this concept by demonstrating that such taxa are not acting in isolation but are embedded within highly interconnected metabolic networks, amplifying their functional impact. This systems-level perspective is consistent with recent microbiome studies emphasising functional redundancy and network behaviour rather than single-organism effects (Birder and Kullmann, 2018; Lane et al., 2022). A key contribution of this study is the identification of two distinct dysbiotic states within NB, a hyperactive metabolic state (Subgroup A) and a depleted functional state (Subgroup B). While previous microbiome studies in urinary dysbiosis have reported variability in microbial composition, few have provided functional differences to interpret this heterogeneity (Chen et al., 2025; Li et al., 2025). Our findings suggest that NB is not a uniform condition but rather exists along a functional continuum characterised by differential metabolic output.

Subgroup A exhibited increased pathway connectivity and metabolic activity, particularly in glycolysis and amino acid metabolism, indicating a metabolically active microbiome capable of producing neuroactive compounds. This may explain clinical phenotypes associated with urgency and detrusor overactivity. In contrast, Subgroup B showed reduced connectivity and pathway activity, reflecting a loss of functional capacity. This aligns with clinical presentations of poor bladder contractility and increased infection risk. Such a dichotomy parallels findings in gut microbiome research, where both hyperactivity and depletion states have been associated with disease (Forster et al., 2020; Gheinani et al., 2025). Healthy controls demonstrated a balanced and distributed network, indicative of functional resilience and homeostasis. In contrast, NB subgroups exhibited either hyperconnectivity (Subgroup A) or network fragmentation (Subgroup B). These findings are consistent with ecological theories of dysbiosis and align well with Anna Karenina’s principle, where disease states are characterised by loss of network stability and dominance of specific functional hubs.

The identification of core pathways in predictive functional analysis includes CoA biosynthesis, phospholipid metabolism, and aromatic compound metabolism as key discriminators, suggesting that central metabolic processes drive disease-associated changes. These pathways are fundamental to microbial survival and host interaction, indicating that NB-associated dysbiosis involves systemic metabolic reprogramming rather than isolated pathway perturbations (Liu et al., 2022; Romano et al., 2025; Wang et al., 2024). Moreover, SHAP-based interpretability analysis provided insights into directional changes, distinguishing between predictive enrichment of pathways in hyperactive states and depleted pathways in functionally impaired states (Chen et al., 2024; Liu et al., 2023). This enhances the translational potential of our findings by identifying candidate biomarkers and therapeutic targets. These findings provide a mechanistic rationale for existing therapies, such as anticholinergics, which may indirectly modulate microbial–neurotransmitter interactions by altering bladder dynamics. Furthermore, the identification of predictive neurotransmitter-related pathways opens avenues for microbiome-targeted interventions, including probiotics, prebiotics, or metabolite-based therapies aimed at restoring neurochemical balance. Similar approaches have shown promising results in gut–brain axis disorders and may apply to urinary tract conditions (Araklitis et al., 2020; Shimizu et al., 2023; Sun et al., 2023; Tan et al., 2024).

Importantly, the strong associations between microbial genera and predictive neurotransmitter-related pathways support the concept of a bladder–brain axis analogous to the gut–brain axis (Shi et al., 2024a; Wang et al., 2025; Yi et al., 2025). While this concept remains underexplored, recent studies have suggested that microbial metabolites in the urinary tract can influence local neuronal signalling and immune responses (Funada et al., 2023; Lucas, 2019). Our study provides predictive functional evidence supporting this hypothesis and highlights the need for further mechanistic validation. ML analysis further demonstrated that both taxonomic and predictive functional features effectively discriminated NB from healthy controls, with the combined model achieving performance of AUC =0.981. This is consistent with previous microbiome-based classification studies, where integration of multi-omics features improves predictive accuracy.

Although the combined ML model demonstrated excellent discrimination by integrating taxonomic and PICRUSt2-predicted functional features, the relatively modest cohort size and overlap between disease subgroups limited multiclass classification; therefore, these findings should be considered exploratory. Future studies should validate these models in larger, independent cohorts and adopt multimodal artificial intelligence frameworks integrating microbiome data with urodynamic parameters, renal imaging, metabolomics, and longitudinal clinical information to improve diagnostic accuracy, biological interpretability, and clinical applicability in paediatric NB (Airoldi et al., 2025; T. Jiang et al., 2026).

Despite these findings, several limitations should be acknowledged. The NDI is an exploratory composite metric based on biologically defined neuroprotective and stress-associated pathways derived from published evidence incorporated into the present dataset and should therefore be regarded as a hypothesis-generating tool rather than a validated clinical biomarker. In addition, the functional profiles were inferred using PICRUSt2 from 16S rRNA sequencing data and therefore represent predicted metabolic potential rather than direct microbial activity. As PICRUSt2 relies on reference genome databases, the inferred pathways should be interpreted cautiously and require validation using shotgun metagenomics, metatranscriptomics, and metabolomics. Nevertheless, PICRUSt2 has been increasingly applied to investigate non-intestinal microbiomes and emerging multi-system microbial axes, including the gut–bladder, gut–brain, gut–skin, and gut–vaginal axes, supporting its utility as a robust hypothesis-generating approach for functional microbiome studies (Akagawa et al., 2024; Chen et al., 2025; Li et al., 2025; Pham et al., 2025; Shi et al., 2024a). Furthermore, the cross-sectional study design and relatively modest cohort size limit causal inference and generalisability. Future studies involving larger, independent cohorts together with shotgun metagenomics, metatranscriptomics, metabolomics, and longitudinal clinical follow-up are required to validate the predicted functions, refine the NDI metrics, and establish causal relationships between microbial metabolism and bladder neural function (W. Chen et al., 2024; Strandwitz, 2018; Sun et al., 2023).

To our knowledge, this is the first study in paediatric NB to integrate urinary microbiome-derived neuroactive functional profiling, the NDI, and ML for disease characterisation. These changes involve coordinated shifts in neurotransmitter-related pathways, metabolic networks, and microbial interactions, which collectively influence bladder function. The integration of microbiome and ML approaches provides both mechanistic insight and diagnostic potential, advancing our understanding of the bladder–brain axis and opening new avenues for targeted therapeutic interventions.

## Data Availability

The metagenomic sequencing data (Fastq files) have been deposited in NIH Sequence Read Archive (SRA) under the BioProject number PRJNA1511179. Additionally all R codes used for data analysis is available on GitHub at https://github.com/drsachitanand/NeurogenicBladder2, ensuring complete transparency.

## Supplementary material

Supplementary Figure 1 Neuroactive Taxa–Pathway Associations Stratified by Group Hierarchical clustering heatmap depicting the strength of association between urine microbial genera (*Collinsella*, *Lactobacillus*, *Acinetobacter*, *Corynebacterium*, *Staphylococcus*, *Veillonella*, *Enterococcus*, *Streptococcus*, *Bifidobacterium*, *Escherichia-Shigella*) and multiple neuroactive metabolic pathways. Each column represents a specific pathway–group combination (e.g., PWY-7220_A, GLUCONEO-PWY_B), and the colour scale ranges from (+3, strong positive association) to (−3, strong inverse association), with white indicating no association. The dendrogram on both axes reflects hierarchical clustering of taxa and pathway–group combinations. Notably, a distinct cluster of Group A–specific pathways shows strongly enriched positive associations with *Collinsella* and *Lactobacillus*. At the same time, *Acinetobacter*, *Corynebacterium*, and *Staphylococcus* display broad negative associations, particularly in Group B and C pathway combinations.

Supplementary Figure 2 ROC Curve Comparisons for Stratified Classification, Random Forest Model Performance and Feature Importance, and RF Proximity MDS Plots for Sample Clustering in NB **(a)**. Receiver Operating Characteristic (ROC) curves comparing the discriminative performance of three feature sets in classifying Control vs. Diseased subjects: Pathways alone (AUC = 0.882), Genus-level microbial features alone (AUC = 0.98), and a Combined model integrating both (AUC = 0.981). The diagonal grey line represents chance-level classification (AUC = 0.5). The Combined and Genus models achieve near-identical and superior performance over pathway-only features, indicating that microbial taxonomic composition is the primary driver of classification accuracy, whilst pathways provide complementary but marginal additional information. **(b)**. ROC curves comparing three Random Forest (RF) classification models: Full RF incorporating all features (AUC = 1.0), Neuroactive-only RF (AUC = 0.882), and Genus + Pathway RF (AUC = 0.984). The Full RF achieves perfect discrimination (AUC = 1.0), indicating overfitting or complete separability with the full feature set. The Genus + Pathway RF closely approximates perfect performance, whilst the Neuro-only RF, despite a lower AUC, still demonstrates strong classification ability, confirming that neuroactive pathway features alone carry substantial discriminative information for disease classification. **(c)**. RF feature importance for the top 15 neuroactive pathways, ranked by Mean Decrease in Gini impurity. PHOSLIPSYN-PWY, COA-PWY-1, and TRNA-CHARGING-PWY are the most important features, followed closely by COA-PWY, COMPLETE-ARO-PWY, PWY-5659, and ARO-PWY. Additional pathways, including HISDEG-PWY, PWY-5189, GLUTORN-PWY, FOLSYN-PWY, and PWY-5100, contribute moderately. FERMENTATION-PWY has the lowest importance amongst the listed pathways. The consistent presence of aromatic amino acid synthesis, CoA, and tRNA-related pathways across the top ranks underscores their centrality in defining the neuroactive microbiome signature associated with disease. **(d)**. One-vs-Rest multiclass ROC curves evaluating the ability of the classification model to distinguish each group separately from the remaining two: Control/C (AUC = 0.698), Disease A (AUC = 0.692), and Disease B (AUC = 0.721). All three curves follow a stepped pattern with only moderate performance, reflecting the challenge of discriminating amongst three overlapping disease subgroups. Disease A achieves slightly higher AUC, suggesting it is marginally more separable from the combined Control and Disease B population than the other classes. **(e)** Multi-Dimensional Scaling (MDS) plot of Random Forest proximity scores for binary classification (Control vs. Diseased). Each point represents a subject (blue = Control, red = Diseased), and distance reflects RF-derived similarity based on how frequently samples co-occur in the same terminal nodes. Confidence ellipses define the 95% boundary for each group. While there is visible separation along MDS1, the two ellipses substantially overlap, indicating partial but incomplete separation of Control and Diseased subjects in RF proximity space, consistent with a moderate but not perfect classification boundary. **(f)** Random Forest Proximity MDS plot for the three-class comparison (Control/C = blue, Group A = green, Group B = red), with MDS1 explaining 75.1% and MDS2 explaining 24.3% of the variance in proximity distances. Elliptical decision boundaries for each group are overlaid. The three groups overlap considerably, particularly along the MDS2 axis, with Group B and Control showing the greatest spatial separation. Group A occupies an intermediate position between Control and Disease B, further illustrating the biological continuum amongst disease subgroups and the inherent challenge of multi-class discrimination.

Supplementary Figure 3 Class-Specific Neuroactive Pathways Driving RF Classification A three-panel horizontal bar chart illustrating class-specific importance scores for neuroactive pathways in the Random Forest classifier, separately for Disease Group (A), Group (B) and Control Group (C). Each panel presents the top 10 pathways with the highest class-specific importance for that group. Group A is primarily characterised by GLUDEG-I-PWY and PWY-5100 as top contributors, with modest scores across LIPASYN-PWY, ORNDEG-PWY, and POLYAMSYN-PWY. Group B is distinguished by ORNDEG-PWY with the largest score, followed by FERMENTATION-PWY, MET-SAM-PWY, and PWY-5188, whilst HISDEG-PWY has the smallest Group B-specific importance. Group C (Control) is dominated by TRNA-CHARGING-PWY and PHOSLIPSYN-PWY with the highest scores (~11–12), followed closely by ARO-PWY, COMPLETE-ARO-PWY, COA-PWY-1, COA-PWY, and GLUTORN-PWY.
